# Incidence of and risk factors for stoma‐site incisional herniation after reversal

**DOI:** 10.1002/bjs5.50165

**Published:** 2019-05-07

**Authors:** A. J. Brook, F. De Haes, N. J. Smart, S. D. Mansfield, I. R. Daniels

**Affiliations:** ^1^ Exeter Surgical Health Service Research Unit Royal Devon and Exeter Hospital Barrack Road, Exeter EX2 5DW UK

We read with interest the work of Amelung and colleagues, recently published in the journal^1^. This retrospective cohort identified several factors associated with increased hernia risk following ostomy closure. However, we identified considerable heterogeneity within the groups included in their analysis, particularly patients with hernias at the site of both ileostomies and colostomies, and variable indications for their construction. It is notable that twice as many patients with a colostomy presented with hernia compared with those with an ileostomy. The technique of surgical closure was not standardized, leading to potential confounding effects of methods, sutures and technique. To gain a proper understanding of risk factors for stoma‐site hernia development, we suggest more robust stratification of these factors. Perhaps the data could be analysed to compare the factors associated with stoma‐site hernia by stoma type reversed.

It is interesting that hypertension is associated with abdominal wall failure, a relationship previously identified in our own work following loop ileostomy closure^2^. The significance of BMI and hypertension appear to be overtaking smoking as a risk factor, as smoking was not a significant factor in this analysis or others^2^, ^3^. Optimizing BP and weight reduction are logical ways to reduce the incidence of hernia, as well as the selective use of prophylactic mesh implants in high‐risk patients. Further work is required to assess mechanisms related to hernia recurrence and hypertension. Mounting evidence now suggests the main predictor of failure is the patient, not the surgeon, and, of course, both parties can work together to reduce risks.

## Disclosure

The authors declare no conflict of interest.
